# Extraction of ingredients from tea leaves using oxidative enzymatic reaction and optimization of extraction conditions

**DOI:** 10.1038/s41598-021-83232-x

**Published:** 2021-02-18

**Authors:** Rasool Pelalak, Afrasyab Khan, Masoud Habibi Zare, Mohammad Hasan Sadeghi, Azam Marjani

**Affiliations:** 1 Institute of Research and Development, Duy Tan University, Da Nang, 550000 Vietnam; 2 Faculty of Environmental and Chemical Engineering, Duy Tan University, Da Nang, 550000 Vietnam; 3 Department of Hydraulics and Hydraulic and Pneumatic Systems, Institute of Engineering and Technology, South Ural State University (SUSU), Lenin Prospect 76, Chelyabinsk, Russian Federation 454080; 4 Department of Chemical Engineering, Isfahan University of Technology, 84156-83111 Isfahan, Iran; 5 Department for Management of Science and Technology Development, Ton Duc Thang University, Ho Chi Minh City, Vietnam; 6 Faculty of Applied Sciences, Ton Duc Thang University, Ho Chi Minh City, Vietnam

**Keywords:** Biocatalysis, Enzymes

## Abstract

Peroxidase (POD) and polyphenol oxidase (PPO) are used as biocatalyst in many processes such as oxidization reactions, wastewater treatment, phenol synthesis and so on. The purpose of current study is enzymes extraction from biomass (tea leaves) as well as evaluation of their activation. Different parameters including temperature, buffer concentration, buffer type, buffer/tea leaves ratio, addition of high molecular weight polymers and emulsifiers, and pH were optimized in order to obtain the highest enzymes activity. Response Surface Methodology (RSM) procedure is employed for statistical analysis of enzymes extraction. It is understood from the result that PPO and POD possess the highest activity at temperatures of 25 °C and 50 °C, pH 7 and 5, buffer molarity of 0.1, and 0.05, buffer/tea leaves ratio = 5 for both, contact time = 20 min and 10 min, and presence of 6% and 3% PVP, 5% and 0% *Tween 80* for PPO and POD, respectively. Amounts of highest activity for PPO and POD biocatalysts were calculated 0.42 U/mL and 0.025493 U/mL, respectively. Moreover, the entire inactivation of PPO took place after 30 min at 40 °C and 60 °C and 20 min at 80 °C. However, POD lost 35% of its activity after 30 min at 40 °C and 60 °C. The amount of 6% POD activity was kept after 45 min at 80 °C. Generally, it was indicated that POD was more resistant to thermal treatment than PPO.

## Introduction

Extraction of enzyme from various sources and its application as a biocatalyst is one of the important achievements in the field of biochemical industry^1–12^. PPO enzyme is known one of the most prominent copper-containing biocatalysts possessing a great potential to catalyse two various reactions consisting of (1) o-diphenols to o-quinones oxidation and molecular oxygen, and (2) the o-hydroxylation of monophenols to o-diphenols^13,14^. This enzyme plays an indisputable role in the appropriate performance of different organs of human body (i.e. eye/skin melanization), and browning the agricultural crops (i.e. fruits)^15–17^. POD is able to be extensively used in various applications such as water/wastewater industry (i.e., wastewater treatment), phenolic components' degradation process, and medical-related kits to diagnose diseases. The classification of POD biocatalyst into classes I, II and III may be done according to its existing sources^18,19^. PPO is existed in different amounts in living organisms (i.e., fungi, insects and mammals). The aforementioned biocatalyst is localized freely in the cytosol in plants^20^. POD is regarded as an important biocatalyst, which its presence/activity plays a momentous role in the lignification process of plants^17,18,21^.

Extraction, purification and characterization process of PPO and POD has been of tremendous attention in disparate agricultural crops (i.e. vegetables) because of the incontrovertible significance of enzymatic browning in food technology. Various advanced separation techniques have been recently developed for chemical/biochemical applications including membranes^22–29^, adsorption^30–37^, and solvent extraction^38^. The mentioned methods can be utilized for extraction of enzymes. In a research, Khajeh et al. developed an instant method to pre-concentrate the trace amount of methylene blue from water using silver nanoparticles. They proved that considering the optimum operational conditions, the detection limit, relative standard deviation and the linear range were achieved 15.0 mg L^−1^, less than 2.7% and between 0.07 and 1.0 mg L^−1^ with a R-squared of 0.996, respectively^39^. Jain et al. experimentally investigated the amount of POD extraction from potato and cabbage applying supercritical CO_2_. They concluded that the extraction process of POD at a specific operational condition (T = 40 °C and P = 10 MPa) is considerably encouraging for use in remediation^40^. Panadare et al. applied the three-phase partitioning procedure with the aim of evaluating the extraction trend of the POD from bitter gourd waste. To do this, they used dimethyl carbonate as an organic phase. They have concluded from their investigation that the purity of POD biocatalyst was 4.84 fold at optimum source concentration (0.15 g mL^−1^)^41^. Khajeh and Gharan applied the zinc oxide nanoparticles–chitosan as an adsorbent to extract organic compounds (i.e. citric and tartaric) from biological samples. They corroborated that the optimum process conditions could be occurred when the pH value of solution = 10, adsorbent mass = 0.5 g, eluents volume = 2.5 mL, sample flow rate = 2 mL min^−1^ and eluent flow rate = 3 mL min^−1^^42^.

Reverse micelles method is known as a noteworthy technique, which is widely employed to extract PPO biocatalyst from apple skin^43^. Batista et al. conducted an experimental investigation to assess the extraction efficiency of PPO biocatalyst from ripe and unripe fruits such as *Solanum lycocarpum.* They perceived the superiority of unripe fruits in terms of activity compared to ripe fruits^15^. Up to the knowledge of the authors, very few papers evaluated the extraction as well as statistical analysis of PPO and POD biocatalysts from green tea as biomass. Therefore, future investigations about this scope seem to be necessary and beneficial for valorization of biomass^17^.

The principal objective of this article is to evaluate the extraction amount of POD and PPO enzymes from Iranian tea leaves accompanying with studying the analysis and assessment of activity at disparate operational circumstances. Optimization of the extraction procedure to achieve pure enzymes is performed using RSM technique. This research paper is the molecular extraction of enzyme. Due to the existence of different variables including Temperature, Buffer pH, Buffer to tea leave ratio, extraction time, Buffer molarity, *PVP* content, and *Tween 80* concentration, this work can be regarded as one of the most complex experimental designs in the field of extraction, which has been done with great success to improve the process efficiency.

## Results and discussion

### Influence of the solvent type

**Figure 1:**
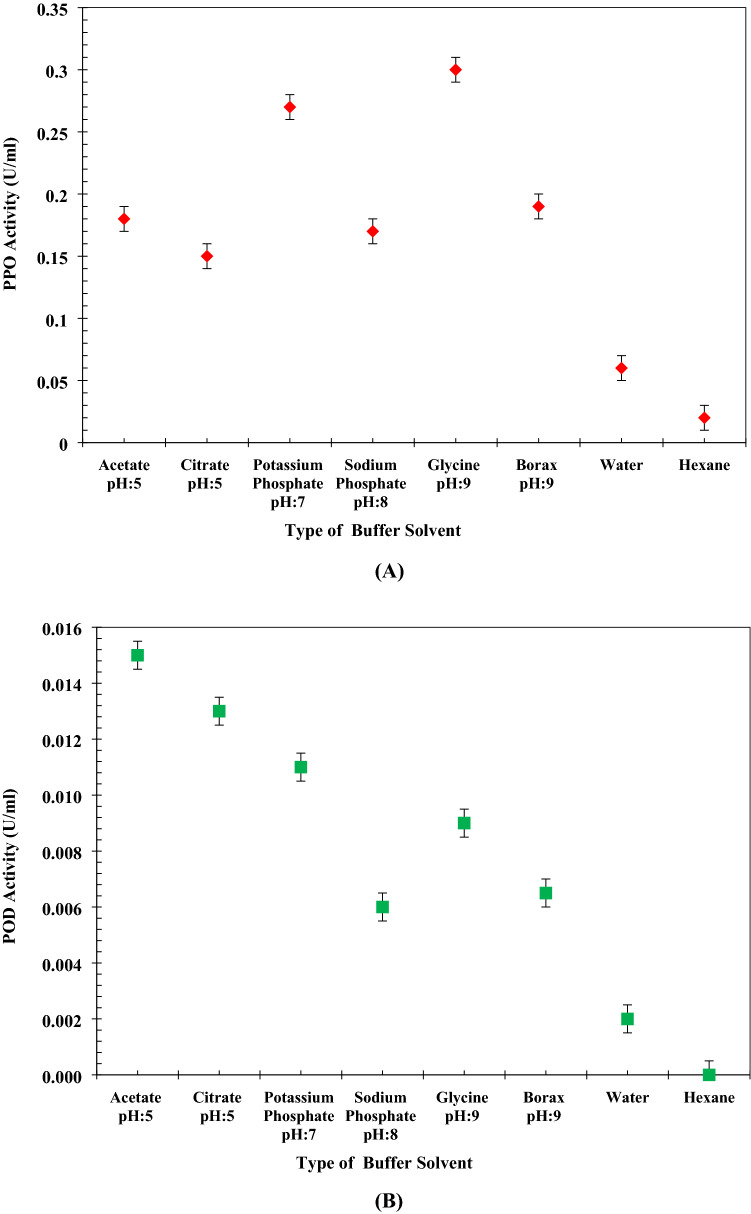
Influence of buffer solvent type on (**A**) PPO activity (**B**) POD activity.

The influence of some momentous parameters such as buffer type, water, and hexane on the performance of PPO and POD extraction in the pH values between 4 to 9 is illustrated in Fig. 1A and B. It is obvious from the results that by applying glycine and acetate as chemical solvents, the highest amount of activities of PPO and POD (0.3 and 0.015 U/ml) was achieved. As can be seen in Fig. 1B, a general decrement in the activity of POD takes place, which is justified due to the application of buffer solutions with greater pH value. Moreover, by using non-buffer solutions as solvent, the activity of both PPO and POD biocatalysts declines considerably. Based on the observations, the authors found out that the utilization of buffer to extract PPO and POD biocatalysts seems to be more reasonable. Significant sensitivity of biomaterials to hydrogen/hydroxide ions is considered as the second justification of using buffer. The occurrence of reaction between the aforementioned ions negatively affects the structure of proteins and biomaterial. Additionally, it is observed from the figures that the amount of activity for PPO is almost 10 times POD.

### Influence of operating conditions on biocatalysts activity

Response level Methodology (Box-Behnken) is employed in this article to achieve the optimum circumstances for 7 parameters through a set of minimum required experiments. The implemented DoE (Design of Experiment) was performed on the basis of Box-Behnken method. The application of abovementioned technique eventuates in decreasing the number of experiments from 152 to 62. Table 1 renders the detailed specifications of the experiments and enzymes activity design based on Box-Behnken method. It is seen from the table that the highest amounts of PPO and POD activities are 0.42 and 0.025493 U/mL, respectively.

**Table 1:** The detailed specifications of the experiments and PPO/POD activity design based on the Box-Behnken method.

Run	T (°C)	pH	L/S	t (min)	Buff. Conc. (M)	PVP (%)	Tween 80 (%)	Response 1	Response 2
	X1	X2	X3	X4	X5	X6	X7	PPO activity (U/ml)	POD activity (U/ml)
1	0	0	−1	−1	0	0	1	0.205	0.012057
2	0	0	0	0	0	0	0	0.296	0.017416
3	0	0	0	−1	1	1	0	0.323	0.018986
4	−1	0	0	0	0	−1	1	0.311	0.018278
5	1	0	0	0	0	−1	1	0.310	0.018239
6	0	0	0	−1	−1	−1	0	0.278	0.016344
7	0	0	−1	1	0	0	−1	0.162	0.009531
8	−1	0	0	0	0	1	1	**0.420**	0.024689
9	0	0	0	0	0	0	0	0.319	0.018756
10	0	1	1	0	0	1	0	0.273	0.016077
11	1	1	0	1	0	0	0	0.217	0.012766
12	0	−1	0	0	1	0	−1	0.287	0.016861
13	−1	0	1	0	1	0	0	0.244	0.014354
14	1	−1	0	−1	0	0	0	0.392	0.023062
15	0	1	0	0	−1	0	1	0.236	0.013856
16	0	0	0	−1	1	−1	0	0.268	0.015751
17	0	0	0	1	1	−1	0	0.278	0.016344
18	0	−1	1	0	0	−1	0	0.248	0.014584
19	0	0	0	1	−1	−1	0	0.276	0.016249
20	0	0	−1	−1	0	0	−1	0.286	0.016823
21	−1	0	0	0	0	1	−1	0.410	0.024096
22	−1	0	−1	0	−1	0	0	0.231	0.013569
23	0	−1	1	0	0	1	0	0.326	0.019158
24	0	0	0	−1	−1	1	0	0.369	0.021684
25	0	0	1	−1	0	0	−1	0.250	0.014718
26	1	−1	0	1	0	0	0	0.334	0.019636
27	0	−1	0	0	−1	0	0	0.365	**0.025493**
28	0	1	0	0	1	0	−1	0.292	0.017167
29	0	0	0	1	−1	1	0	0.376	0.022124
30	0	1	0	0	−1	0	−1	0.340	0.019981
31	0	−1	−1	0	0	−1	0	0.195	0.011445
32	0	−1	0	0	−1	0	1	0.360	0.021187
33	0	0	1	1	0	0	−1	0.242	0.014258
34	0	0	0	0	0	0	0	0.303	0.017837
35	−1	0	−1	0	1	0	0	0.228	0.013416
36	−1	0	1	0	−1	0	0	0.238	0.014010
37	−1	1	0	−1	0	0	0	0.270	0.015885
38	−1	0	0	0	0	−1	−1	0.306	0.017990
39	0	1	−1	0	0	1	0	0.245	0.014431
40	1	0	1	0	1	0	0	0.256	0.015081
41	0	−1	−1	0	0	1	0	0.292	0.017148
42	1	0	−1	0	−1	0	0	0.214	0.012612
43	0	0	0	0	0	0	0	0.299	0.017608
44	−1	−1	0	1	0	0	0	0.394	0.023158
45	1	1	0	−1	0	0	0	0.338	0.019904
46	0	0	1	−1	0	0	1	0.177	0.010392
47	1	0	0	0	0	−1	−1	0.294	0.017301
48	−1	−1	0	−1	0	0	0	0.250	0.014718
49	0	0	0	0	0	0	0	0.304	0.017876
50	0	1	1	0	0	−1	0	0.172	0.010144
51	0	0	−1	1	0	0	1	0.235	0.013837
52	0	0	0	0	0	0	0	0.307	0.018086
53	0	1	0	0	1	0	1	0.306	0.017971
54	1	0	1	0	−1	0	0	0.291	0.017091
55	1	0	0	0	0	1	−1	0.411	0.024172
56	0	0	0	1	1	1	0	0.380	0.022354
57	0	1	−1	0	0	−1	0	0.157	0.009244
58	1	0	0	0	0	1	1	0.403	0.023732
59	1	0	−1	0	1	0	0	0.204	0.012000
60	0	0	1	1	0	0	1	0.328	0.019311
61	0	−1	0	0	1	0	1	0.388	0.022813
62	−1	1	0	1	0	0	0	0.340	0.020000

Bold values indicate the maximum value.

Influence of each parameter on activity amount of both biocatalysts (PPO and POD) is computed and shown in Table 2. It can be seen that the temperature has the highest impact on the PPO and POD activities and the second order is allocated to the buffer pH of buffer solution. It was also concluded that other parameters such as the buffer-to-mass of tea ratio, contact time, and PVP content don’t have a significant influence on the PPO activity according to the analysis of variance (ANOVA) calculation^17^. Also, The ANOVA fixes the relative effect of error (REE) at 0.03% and up to 0% for PPO and POD, respectively. REE means that the influences of other parameters not included in the analysis and any unintentional discrepancies were considered in optimization.

**Table 2:** Effect of disparate parameters on the PPO and POD activity (ANOVA).

Source	Sum of squares	Df	Mean square	F value	P value	
*Response 1: PPO Activity* (U/ml)						
Model	0.2614	35	0.0075	106.73	< 0.0001	Significant
A-Temperature	0.0000	1	0.0000	35.46	< 0.0001	Significant
B-pH	0.0172	1	0.0172	246.36	< 0.0001	Significant
C-L/S	0.0064	1	0.0064	91.52	< 0.0001	Significant
D-t (min)	0.0010	1	0.0010	14.70	0.0007	Significant
E-Buffer. Conc. (M)	0.0006	1	0.0006	8.68	0.0067	Significant
F-PVP (%)	0.0536	1	0.0536	766.42	< 0.0001	Significant
G-Tween-80 (%)	0.0000	1	0.0000	0.6687	< 0.0001	Significant
AB	0.0023	1	0.0023	33.36	< 0.0001	Significant
AC	0.0014	1	0.0014	19.73	0.0001	Significant
AD	0.0193	1	0.0193	275.94	< 0.0001	Significant
AE	0.0003	1	0.0003	4.09	0.0536	Not significant
AF	8.469E−07	1	8.469E−07	0.0121	0.9132	Not significant
AG	5.293E−06	1	5.293E−06	0.0756	0.7855	Not significant
BC	0.0002	1	0.0002	3.50	0.0728	Not significant
BD	0.0023	1	0.0023	33.36	< 0.0001	Significant
BE	0.0007	1	0.0007	9.57	0.0047	Significant
BF	0.0000	1	0.0000	0.3661	0.5504	Not significant
BG	0.0043	1	0.0043	62.09	< 0.0001	Significant
CD	0.0071	1	0.0071	100.77	< 0.0001	Significant
CE	0.0000	1	0.0000	0.4177	0.5237	Not significant
CF	5.293E−06	1	5.293E−06	0.0756	0.7855	Not significant
CG	0.0001	1	0.0001	0.7269	0.4017	Not significant
DE	0.0005	1	0.0005	6.75	0.0152	Significant
DF	0.0004	1	0.0004	5.66	0.0250	Significant
DG	0.0123	1	0.0123	175.73	< 0.0001	Significant
EF	0.0001	1	0.0001	2.01	0.1685	Not significant
EG	0.0063	1	0.0063	89.77	< 0.0001	Significant
FG	0.0000	1	0.0000	0.5930	0.4482	Not significant
A^2^	0.0040	1	0.0040	56.50	< 0.0001	Significant
B^2^	0.0000	1	0.0000	0.2030	0.6560	Not significant
C^2^	0.0936	1	0.0936	1337.38	< 0.0001	Significant
D^2^	0.0002	1	0.0002	3.08	0.0911	Not significant
E^2^	1.899E−06	1	1.899E−06	0.0271	0.8704	Not significant
F^2^	0.0044	1	0.0044	62.28	< 0.0001	Significant
G^2^	0.0045	1	0.0045	63.70	< 0.0001	Significant
Residual	0.0018	26	0.0001			
Lack of Fit	0.0015	21	0.0001	1.14	0.4858	not significant
Pure Error	0.0003	5	0.0001			
Cor Total	0.2632	61	R^2^			0.9931
Std. Dev	0.0084		Adjusted R^2^			0.9838
Mean	0.2911		Predicted R^2^			0.9576
C.V. %	2.87		Adeq Precision			40.2724
*Response 2: POD Activity* (U/ml)						
Model	1.420E−06	35	4.057E−08	75.20	< 0.0001	Significant
A-Temperature	8.234E−11	1	8.234E−11	15.26	< 0.0001	Significant
B-pH	1.057E−07	1	1.057E−07	195.88	< 0.0001	Significant
C-L/S	3.911E−08	1	3.911E−08	72.50	< 0.0001	Significant
D-t (min)	4.389E−09	1	4.389E−09	8.14	0.0084	Significant
E-Buffer. Conc. (M)	6.298E−09	1	6.298E−09	11.67	0.0021	Significant
F-PVP (%)	2.652E−07	1	2.652E−07	491.60	< 0.0001	Significant
G-Tween-80 (%)	2.785E−10	1	2.785E−10	0.5163	0.4789	Not significant
AB	1.271E−08	1	1.271E−08	23.56	< 0.0001	Significant
AC	8.343E−09	1	8.343E−09	15.47	0.0006	Significant
AD	9.261E−08	1	9.261E−08	171.68	< 0.0001	Significant
AE	1.646E−09	1	1.646E−09	3.05	0.0925	Not significant
AF	1.097E−13	1	1.097E−13	0.0002	0.9887	Not significant
AG	9.456E−12	1	9.456E−12	0.0175	0.8957	Not significant
BC	1.252E−09	1	1.252E−09	2.32	0.1398	Not significant
BD	1.267E−08	1	1.267E−08	23.49	< 0.0001	Significant
BE	1.151E−08	1	1.151E−08	21.33	< 0.0001	Significant
BF	8.905E−10	1	8.905E−10	1.65	0.2102	Not significant
BG	9.584E−09	1	9.584E−09	17.77	0.0003	Significant
CD	4.405E−08	1	4.405E−08	81.66	< 0.0001	Significant
CE	1.170E−10	1	1.170E−10	0.2170	0.6452	Not significant
CF	2.231E−10	1	2.231E−10	0.4136	0.5258	Not significant
CG	6.890E−14	1	6.890E−14	0.0001	0.9911	Not significant
DE	2.098E−09	1	2.098E−09	3.89	0.0594	Not significant
DF	1.580E−09	1	1.580E−09	2.93	0.0989	Not significant
DG	7.768E−08	1	7.768E−08	143.99	< 0.0001	Significant
EF	5.662E−10	1	5.662E−10	1.05	0.3151	Not significant
EG	4.801E−08	1	4.801E−08	88.99	< 0.0001	Significant
FG	2.108E−10	1	2.108E−10	0.3907	0.5374	Not significant
A^2^	1.492E−08	1	1.492E−08	27.65	< 0.0001	Significant
B^2^	2.355E−12	1	2.355E−12	0.0044	0.9478	Not significant
C^2^	5.210E−07	1	5.210E−07	965.83	< 0.0001	Significant
D^2^	2.580E−09	1	2.580E−09	4.78	0.0380	Significant
E^2^	5.933E−10	1	5.933E−10	1.10	0.3039	Not significant
F^2^	1.490E−08	1	1.490E−08	27.62	< 0.0001	Significant
G^2^	2.364E−08	1	2.364E−08	43.83	< 0.0001	Significant
Residual	1.403E−08	26	5.395E−10			
Lack of Fit	1.253E−08	21	5.965E−10	1.99	0.2292	Not Significant
Pure Error	1.499E−09	5	2.999E−10			
Cor Total	1.434E−06	61	R^2^			0.9931
Std. Dev	0.0000		Adjusted R^2^			0.9839
Mean	0.0013		Predicted R^2^			0.9574
C.V. %	1.46		Adeq Precision			40.7835

**Figure 2:**
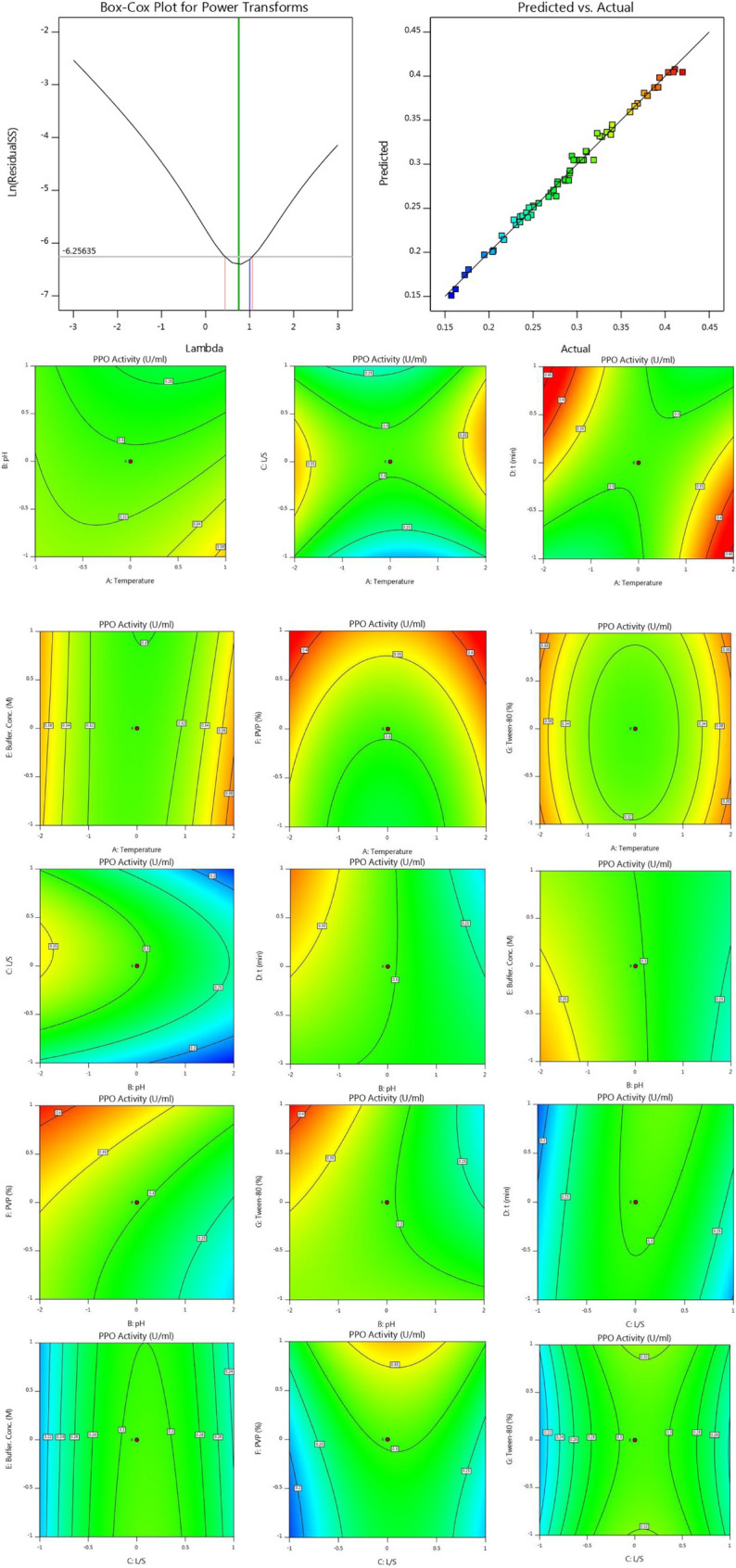

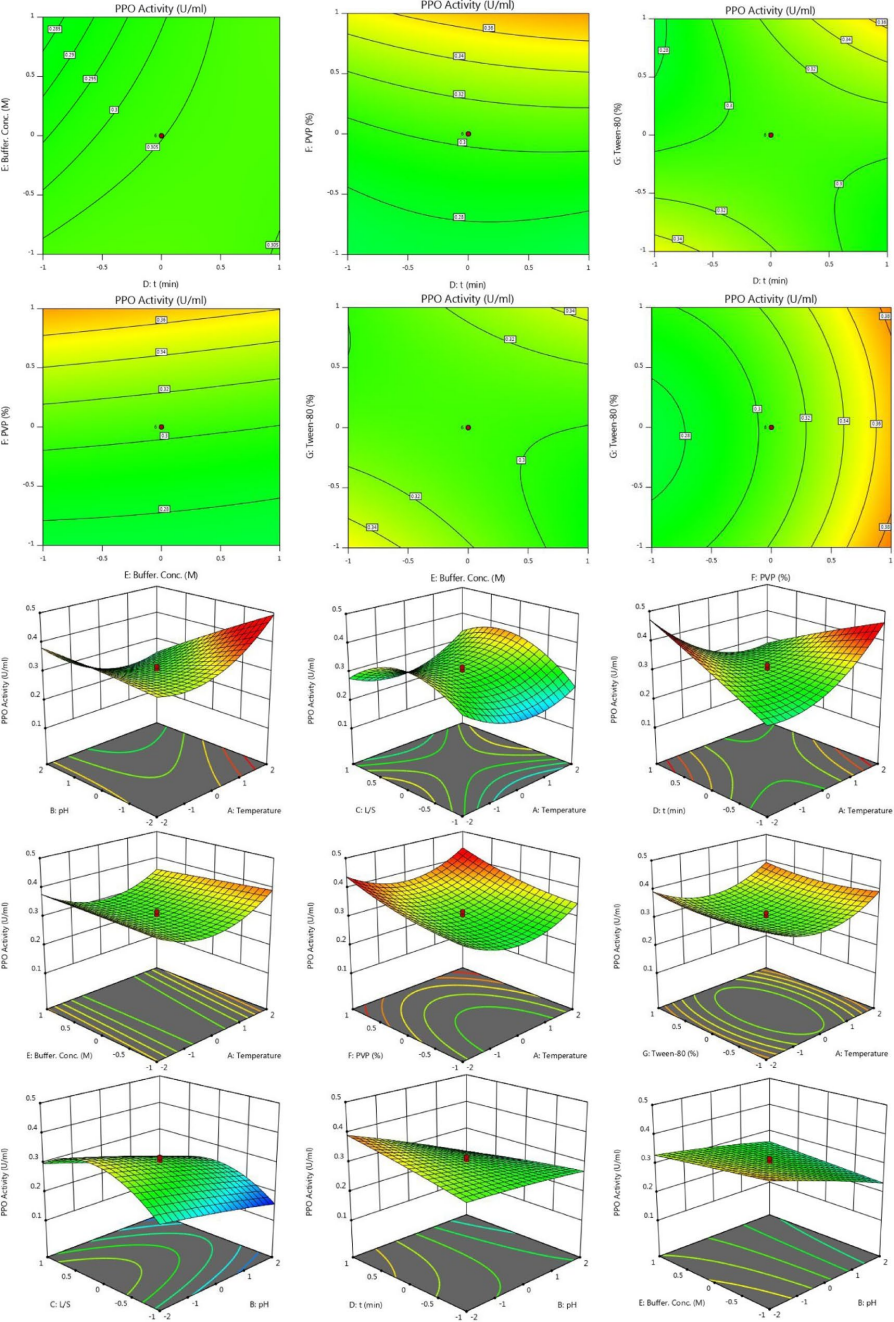

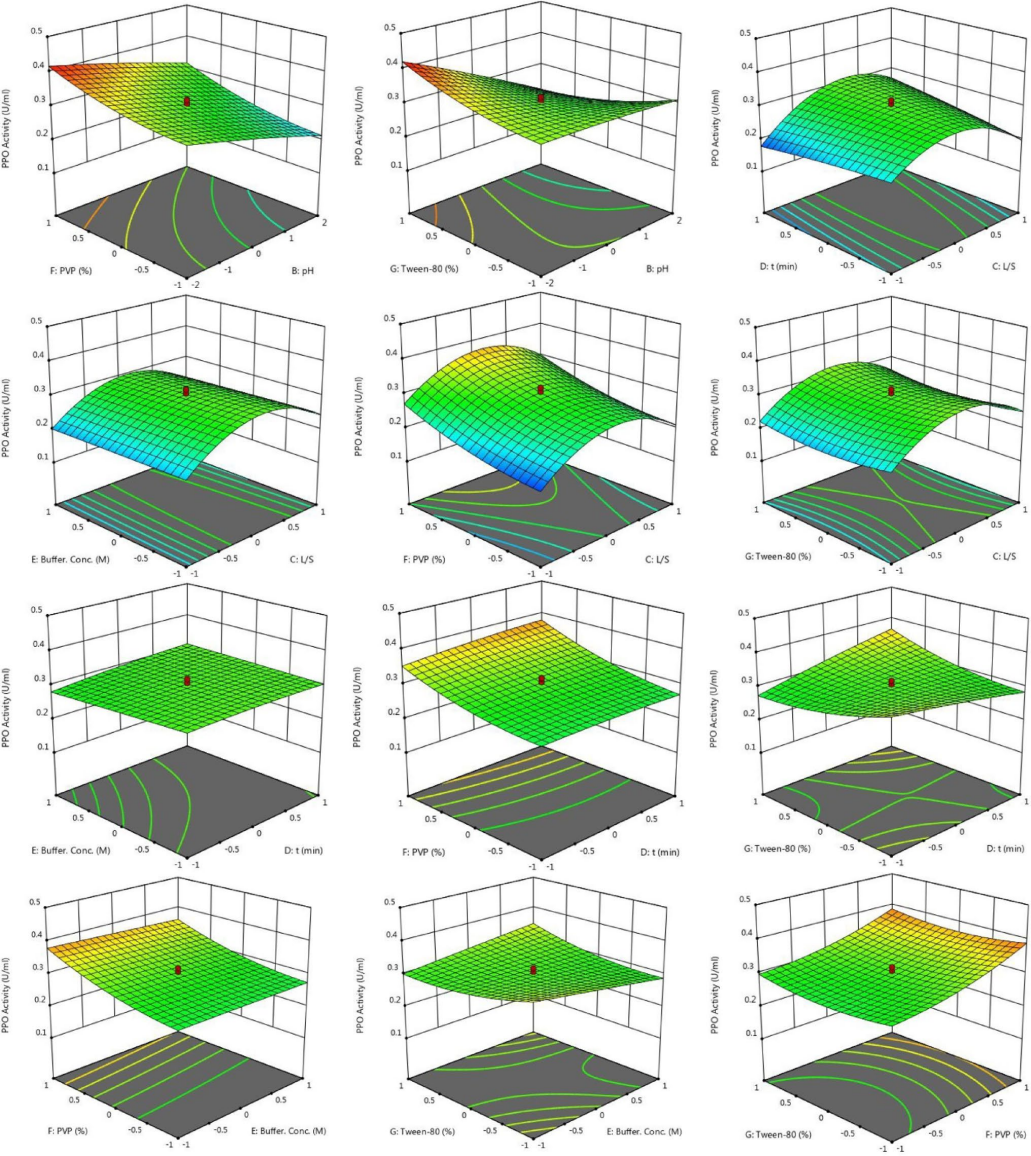
The plot of relationship between predicted and actual values of PPO enzyme activity and Contours of the effect of seven parameters on PPO enzyme activity and 3D response surface plots for the effect of seven parameters on PPO enzyme activity.

**Figure 3:**
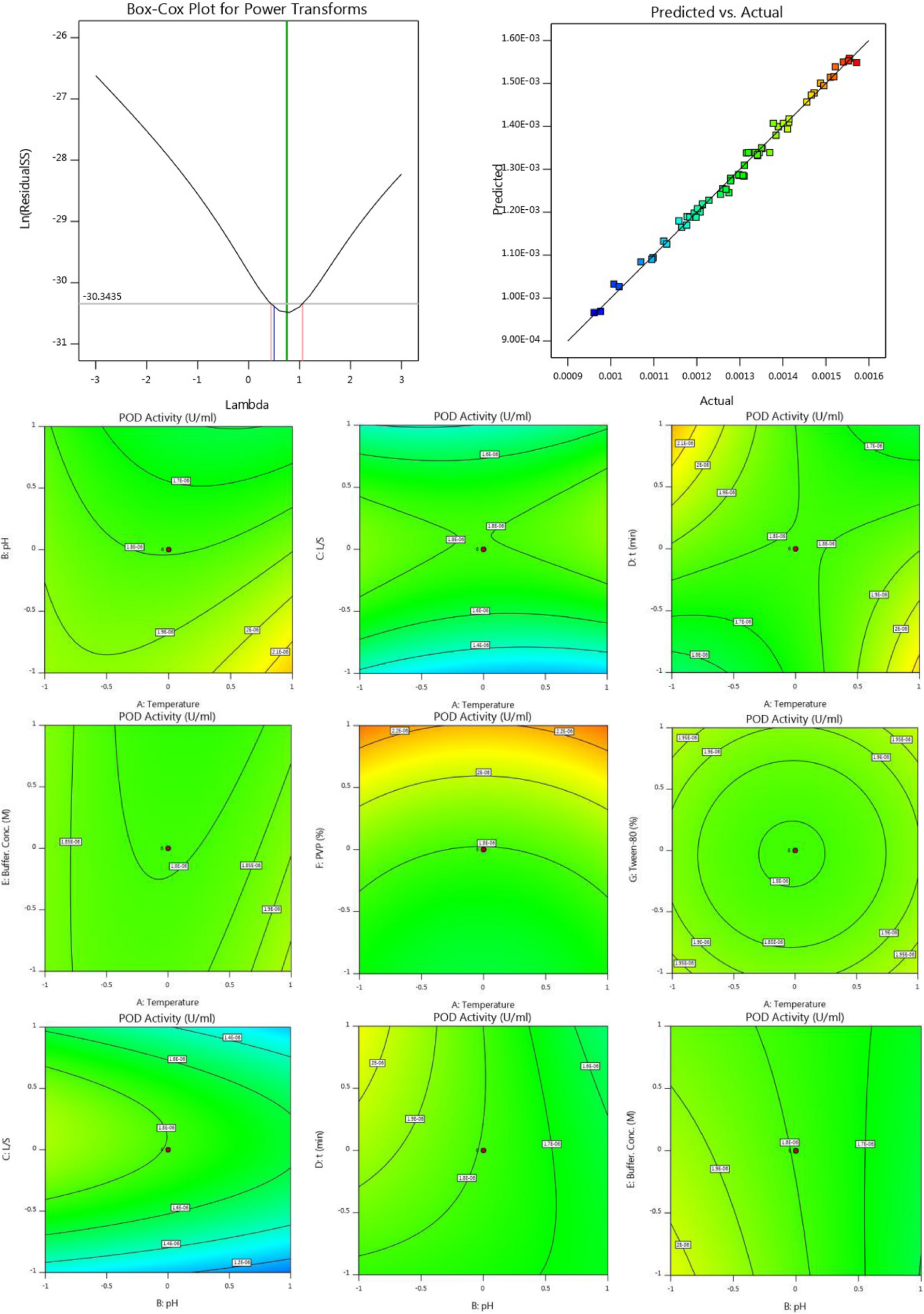

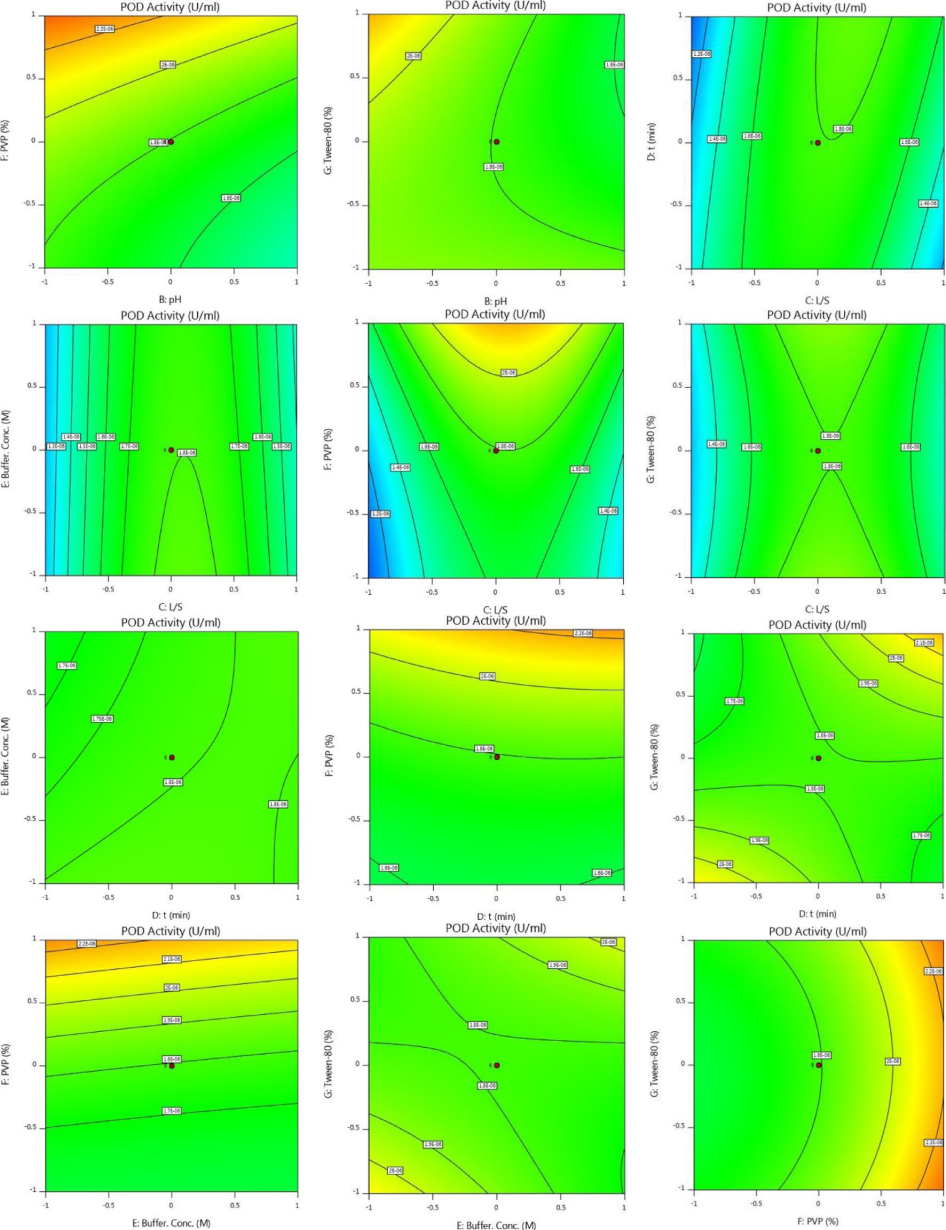

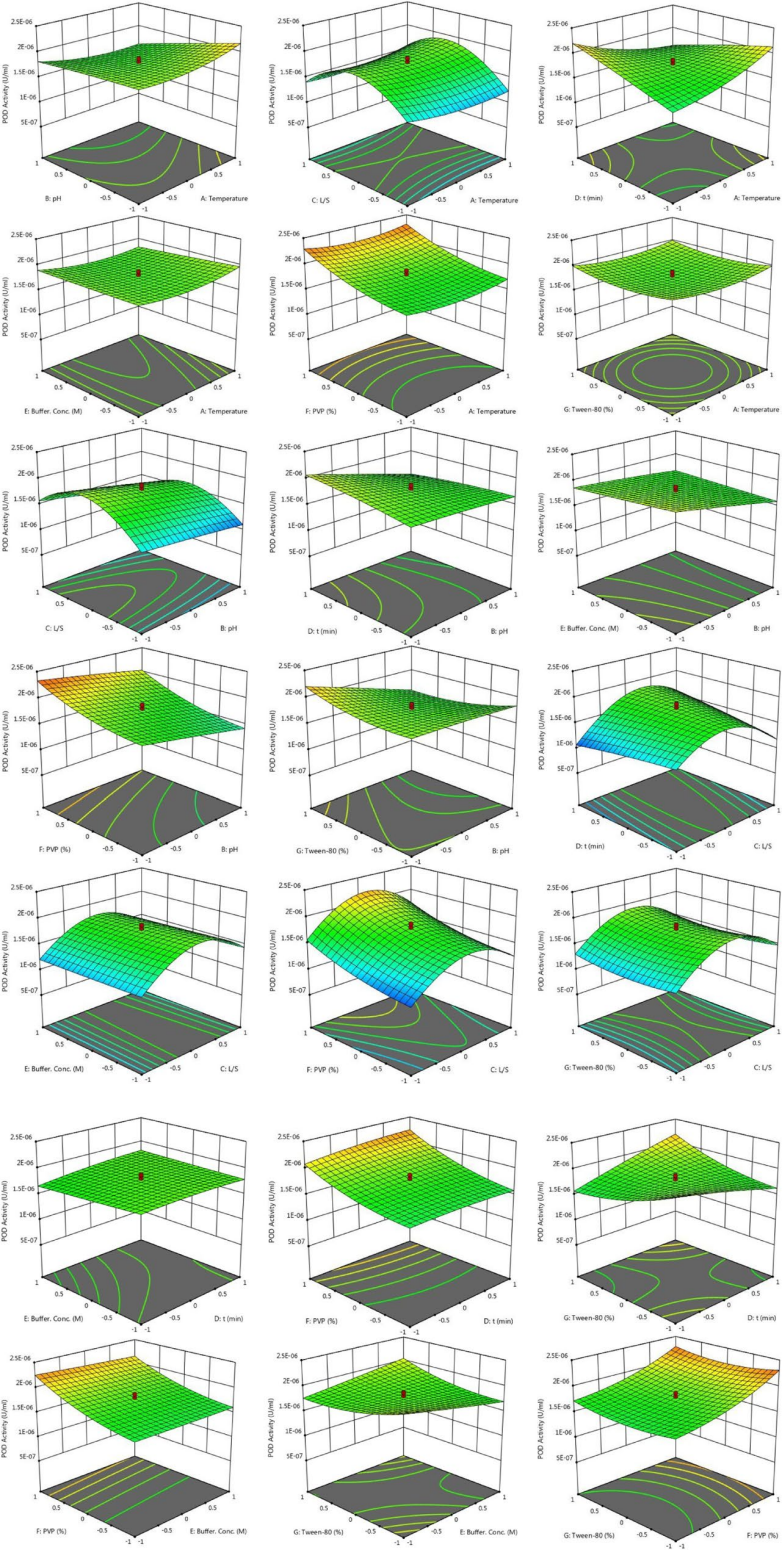
The plot of relationship between predicted and actual values of POD enzyme activity and Contours of the effect of seven parameters on POD enzyme activity and 3D response surface plots for the effect of seven parameters on POD enzyme activity.

According to Response 1, the F-value of 106.73 illustrates the significance of the model. P-values less than 0.05 reveal that the model terms are considered as significant. As such, B, C, D, E, F, AB, AC, AD, BD, BE, BG, CD, DE, DF, DG, EG, A^2^, C^2^, F^2^, G^2^ are known as the important terms of model. Values higher than 0.1 indicate the insignificance of model terms. The Lack of Fit F-value of 1.14 shows that it is not significant related to the pure error. There is a 48.58% chance that a Lack of Fit F-value at this quantity might happen because of noise. The estimated R^2^ value of 0.9576 is in acceptable agreement with the regulated R^2^ of 0.9838 (the difference is lower than 0.2. Adeq precision evaluates the signal to noise ratio. A ratio higher than 4 is favourable. The ratio of 40.272 demonstrates a sufficient signal. This model possesses a great potential to navigate the design space. According to Response 2, the F-value of 75.20 proves the insignificance of the model. There is only a 0.01% chance that an F-value at this quantity might happen because of noise. P-values less than 0.05 illustrate the significance of model terms. In this case, B, C, D, E, F, AB, AC, AD, BD, BE, BG, CD, DG, EG, A^2^, C^2^, D^2^, F^2^, G^2^ are significant model terms. Values higher than 0.1, illustrate that the insignificance of the model. If there are numerous insignificant model terms, then the model reduction can boost the model’s performance. F-value of 1.99 corroborates that the Lack of Fit is not considered as significant related to the pure error^44^. There is a 22.92% chance that a Lack of Fit F-value at this quantity might take place because of noise. Insignificant lack of fit is desirable. The predicted R^2^ of 0.9574 is in acceptable accordance with the regulated R^2^ of 0.9839 (the difference is lower than 0.2). Adeq precision evaluates the signal to noise ratio. A ratio higher than 4 is favourable. The ratio of 40.783 illustrates a sufficient signal. This model is able to be applied to navigate the design space. As shown in Figs. 2 and 3, the results of this experiment are very complex and well illustrated by 7 factors affecting PPO and POD activities. Figures 2 and 3 illustrate the predicted values versus actual values plot for PPO/POD biocatalysts activities. The results indicated an excellent agreement between the model predictions and actual experimental values for both biocatalysts. Therefore, the developed model is confirmed to be an efficacious platform to bridge the correlation between process parameters to the PPO biocatalyst activity. In the continuation of Figs. 2 and 3, all the contours about the relationship of seven factors against each other and ultimately in relation to the activity of the biocatalyst are depicted. These contours appropriately determine the impact of each factor on biocatalyst activity. The impact of temperature on the activity of both PPO and POD biocatalysts was studied at 3 levels (25 °C, 50 °C and 75 °C). It was perceived that both PPO biocatalyst reaches to its maximum activity at 25 °C, while the maximum activity of occurs at 50 °C. As seen in Table 2, the influence of temperature on the extraction and activity of PPO and POD biocatalysts was achieved 55.67 and 22.70%, respectively. Ref.^45^ offered the best temperature of 20 °C for extraction of PPO from lychee^45^. In another study, Yue-Ming et al. reported that the optimum temperature for the PPO activity is about 65 °C^46^. It is able to be found out from the Table 2 that the impact of pH on the extraction and activity of PPO and POD biocatalysts was 24.07 and 47.89%, respectively. The results prove that the increment of pH eventuates in decreasing the activity of PPO and increasing the PPO extraction. However, POD response to pH alteration isn’t desirable, which results in decreasing the POD separation by rising pH of medium. Rudra Shalini understood that the pH values of 7.6 and 6.4 are optimum for PPO and POD activity^47^. Also, the highest amount of PPO activity was in the range of 5–7.5 for the source of bean seeds^48^. It is profoundly believed that tea leaves are considered as an appropriate source for biocatalyst utilization in fundamental media. Biocatalysts (Enzymes) are known as amphoteric molecules, which possess various basic/acidic groups. Alteration of the electric charge of groups is occurred by changing the pH value. It is obvious that changing in the electric charge of groups eventuates in altering the biocatalyst's net charge and its surface charge and subsequently influences the active part of biocatalyst. Overall, the amount of biocatalysts activity around the isoelectric point is maximum and declines gradually when the pH of solution is far from the isoelectric point. The impact of the ratio of buffer to mass of tea was done in three ratios (3, 5, and 7). This parameter varies the amount of PPO/POD activity and extraction by about 0.01%. It would be clear that the effect of buffer-to-mass of tea leaves ratio (L/S) on the POD activity is ignorable. By enhancing the L/S ratio, the activity of PPO biocatalyst increased at first and after that declined. Enhancement of the L/S ratio to an optimum value improves the activity of the biocatalyst. Additionally, increment of the L/S ratio significantly improves mass transfer because of enhancing driving force between two phases^49^. Therefore, increment of L/S ratio may result in the dilution of solution and consequently declining the concentration and activity of biocatalyst in the system. Moreover, the impact of contact between tea leaves and buffer for PPO and POD biocatalysts was achieved 10.50 and 22.17%, respectively. Also, buffer concentration was evaluated in the range of 0.05–0.2 M and it was resulted that it does not have significant influence on the biocatalysts activity. Tea leaves consist of 20–30% various natural polyphenols. Some investigators recommended the addition of PVP into the system to avoid oxidation of these components during extraction process. PVP was applied with the aim of separating polyphenols from biocatalyst solution applying formation of hydrogen bond^17,50^. It can be denoted that the concentration of PVP enhanced from 0 to 6%. Moreover, it is perceived that increase in the PVP concentration improves the activity of PPO/POD biocatalysts. According to ANOVA analysis, the relative impact of PVP addition was achieved 9.08 and 7.13% for PPO and PO, respectively. The exact location of PPO and POD biocatalysts is inside the chloroplast cell. Hence, it is mandatory to apply a surfactant to enhance the activity of enzyme and the efficiency of extraction. By conducting an experimental investigation, Mayer reached to this result that addition of 105 and 15% Triton X-100 eventuated in increasing the extraction efficiency of PPO biocatalyst from apple and tobacco by about 44 and 77%, respectively^51^. In the recent investigation, Tween 80 concentration is applied in the range of 0–20%. According to the obtained data from optimization analysis, it is concluded that the addition of Tween 80 possesses substantial influence on the activity amount PPO and POD. The value of effect was computed 22 and 19% for PPO/POD activity and extraction, respectively. The addition of 5% is considered as the optimum concentration due to the fact that 20% Tween 80 results in producing great amount of in the system^17^.

**Table 3:** Optimized level and value for operation parameters by Design Expert 11.

Parameter	PPO		POD	
	Level	Value	Level	Value
Temperature (°C)	−1	25	0	50
Buffer pH	0	7	−1	5
Buffer to mass of tea ratio	0	5	0	5
Tea and solvent contact time, min	0	10	0	10
Buff. Conc. (M)	0	0.1	−1	0.05
PVP Conc. (%)	1	6	0	3
Tween 80 Conc. (%)	1	20	0	5
Error (%)	0.03		$\cong 0$	

Moreover, Figs. 2 and 3 demonstrate the relationship of seven factors influencing the activity of PPO and POD biocatalysts. The only difference between these two figures is that the connection is displayed in 3D. Due to the complexity of the experiments, this difference helps to have a better understanding of the relationship between the parameters affecting the activity of the two enzymes.

RSM procedure is employed to achieve the optimum conditions for 8 parameters through a set of minimum number of experiments. The optimum operating conditions for extraction of PPO and POD biocatalysts applying the RSM technique are presented in Table 3. The amount of response at optimum conditions is obtained 0.41 U/mL (experimental value = 0.42) and 2.249E−06 U/mL (experimental value = 2.549E−06) for PPO and POD, respectively. It is obvious from the table that RSM procedure can appropriately estimate the optimum conditions particularly for PPO.

### Data fitting for biocatalyst activity

To develop an appropriate regression model for biocatalyst activity, Data Fit 9 software is aimed to be applied. A data fitting is performed for PPO and POD biocatalysts (Eq. 1 for PPO and Eq. 2 for POD) according to the second order equations. The results are presented as follows:

1 $$ \begin{gathered} {\text{PPO}}\;{\text{Activity }}({\text{U/ml}}) = + 0.3048 + \left[ {0.0010*A} \right] - \left[ {0.0268*B} \right] + \left[ {0.0163*C} \right] + \left[ {0.0065*D} \right] \hfill \\ - \left[ {0.0050*E} \right] + \left[ {0.0473*F} \right] + \left[ {0.0014*G} \right] - \left[ {0.0171*A*B} \right] + \left[ {0.0131*A*C} \right] - \left[ {0.0491*A*D} \right] \hfill \\ - \left[ {0.0060*A*E} \right] - \left[ {0.0003*A*F} \right] - \left[ {0.0008*A*G} \right] - \left[ {0.0055*B} \right] \hfill \\ - \left[ {0.0171*B*D} \right] + \left[ {0.0092*B*E} \right] + \left[ {0.0018*B*F} \right] - \left[ {0.0233*B*G} \right] + \left[ {0.0297*C*D} \right] - \left[ {0.0019*C*E} \right] \hfill \\ - \left[ {0.0008*CF} \right] + \left[ {0.0025*C*G} \right] + \left[ {0.0077*D*E} \right] + \left[ {0.0070*D*F} \right] + \left[ {0.0392*D*G} \right] \hfill \\ - \left[ {0.0042*E*F} \right] + \left[ {0.0280*E*G} \right] - \left[ {0.0023* \, FG} \right] + \left[ {0.0171*A^{2} } \right] - \left[ {0.0010*B^{2} } \right] - \left[ {0.0833*C^{2} } \right] - \left[ {0.0040*D^{2} } \right] \hfill \\ - \left[ {0.0004* \, E^{2} } \right] + \left[ {0.0180*F^{2} } \right] + \left[ {0.0182*G^{2} *C} \right] \hfill \\ \end{gathered} $$

2 $$ \begin{gathered} {\text{Sqrt}}\left( {{\text{POD}}\;{\text{Activity}}\;({\text{U/ml}})} \right) = + 0.0013 + \left[ {1.852E - 06*A} \right] - \left[ {0.0001*B} \right] + \left[ {0.00006*C} \right] + \left[ {0.00004*D} \right] \hfill \\ - \left[ {0.000014*E} \right] + \left[ {0.0001*F} \right] - \left[ {3.406E - 06*G} \right] - \left[ {0.00004*A*B} \right] + \left[ {0.000032*A*C} \right] - \left[ {0.0001*A*D} \right] \hfill \\ - \left[ {0.00001*A*E} \right] - \left[ {1.171E - 07*A*F} \right] - \left[ {1.087E - 06*A*G} \right] - \left[ {0.00001*B*C} \right] \hfill \\ - \left[ {0.00004*B*D} \right] + \left[ {0.00003*B*E} \right] + \left[ {0.00001*B*F} \right] - \left[ {0.00003*B*G} \right] + \left[ {0.000074*C*D} \right] \hfill \\ - [3.825E - 06*C*E] - [5.281E - 06*C*F] + [9.281*E] \hfill \\ - \left[ {08*C*G} \right] + \left[ {0.000016*D*E} \right] + \left[ {0.000014*D*F} \right] + \left[ {0.0001*D*G} \right] - \left[ {8.413E - 06*E*F} \right] + \left[ {0.0001*EG} \right] \hfill \\ - \left[ {5.133E - 06*F*G} \right] + \left[ {0.000033*A^{2} } \right] + \left[ {4.176E - 07*B^{2} } \right] - \left[ {0.0002*C^{2} } \right] - \left[ {0.0000*D^{2} } \right] \hfill \\ + \left[ {6.630E - 06*E^{2} } \right] + \left[ {0.000033*F^{2} } \right] + \left[ {0.000042*G^{2} } \right] \hfill \\ \end{gathered} $$

In the case of actual factors, these equations are able to be applied to provide estimations. The computed amounts of activity for PPO/POD biocatalysts considering different operating conditions (62 experiments) applying Eqs. (1) and (2) are provided in Table S1 (Supplementary file). Coefficient of determination (R^2^) was more than 95.76 and 95.74 for PPO and POD biocatalysts, respectively.

### Protein content

In this section, the protein content for PPO and POD biocatalysts at the optimum operating conditions is aimed to be obtained. The protein amount for both abovementioned biocatalysts was achieved 0.26 mg protein/mL. For this purpose, 10 g tea leaves was applied in 25 mL buffer. Hence, it is able to be concluded that there is 0.40 g tea leaves in each mL of buffer solution. According to this observation, 0.46 mg protein is existed in 1 g tea leaves. Calculation of the specific activity is performed according to the protein content. The specific activities of both biocatalysts are computed 3.1 (U/mg protein) and 1.76 (U/g leaves) for PPO and 0.31 (U/mg protein) and 0.083 (U/g leaves) for POD, respectively.

**Figure 4:**
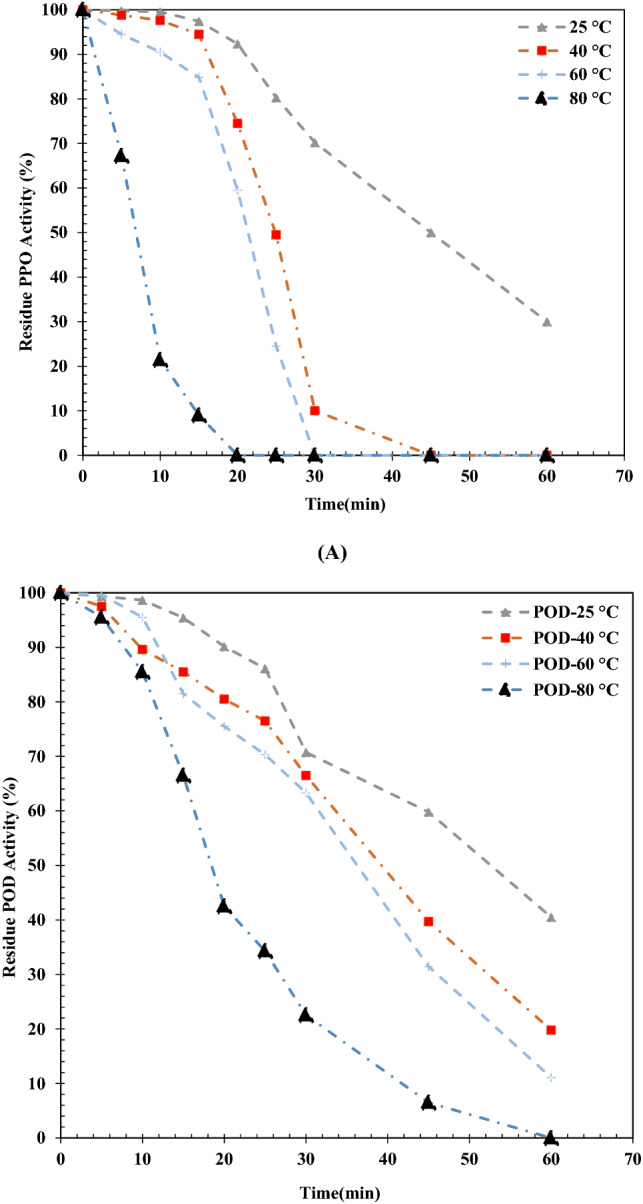
Heat stability of (**A**) PPO as function of time and temperature, (**B**) POD as function of time and temperature.

### Enzymes heat stability

Figure 4A and B demonstrate the heat stability of PPO/POD biocatalysts as a function of time and temperature. It is clear from Fig. 4A that, the amount of PPO biocatalyst activity declines slowly until 15 min when the temperature is 40 and 60 °C, respectively. But, after 15 min, the amount of PPO biocatalyst activity decreases substantially till end time which is half an hour. PPO activity is 10% when the temperature is 40 °C at 30 min and completely inactive at 45 min but it is also completely inactive at 30 min when temperature reaches 60 °C. When the temperature of system is 80 °C, there is a substantial decrement in the amount of PPO biocatalyst activity at initial 20 min it completely becomes inactive after 20 min. There is a moderate slope toward the lower level for POD biocatalyst activity over the entire experiment when the temperature is 40 °C and 60 °C and the POD biocatalyst activity reached 20 and 10%, respectively (60 min). According to the results, the amount of PPO biocatalyst activity is 22% after 30 min when the temperature is 80 °C. The activity of PPO and POD biocatalysts at ambient temperature (25 °C) after 60 min declined to 30 and 40%, respectively. Overally, the heating stability of POD biocatalyst is more than PPO.

## Materials and method

### Preparation of samples

Preparation of Green tea leaves was under the responsibility of Iran Tea Research Centre (Lahijan, Iran). To do this research study, Premier-quality tea leaves were chosen and frozen at a very low temperature (i.e., − 25 °C) until applied. For carrying out the experimental analysis, the lab based-apparatuses including *Domel Tehtnica MillMix* 20, HS 18,500 R laboratory centrifuges, Camspec M350 Spectrophotometer—Manufacturer, digital scale, pH meter, Checker Plus pH Tester with 0.01 pH Resolution-HI98100 were applied^17^. Required chemical materials such as *monopotassium phosphate, catechol, guaiacol, distilled water, hexane, polyvinylpyrrolidone (PVP), hydrogen peroxide* (30%), *sodium hydroxide, acetate, citrate, sodium phosphates, glycine, borax, bovine serum albumin* were supplied from Sigma-Aldrich (St. Louis, MO, USA) and Merck companies (Darmstadt, Germany)^17^. All chemicals were of analytical grade.

### Enzymes extraction

To measure the activity of biocatalysts, their extraction from tea leaves cells seems to be essential. A determined amount of tea leaves was weighed and added into kitchen grinder and was mixed with the solvent. Solvent type is of great importance and buffers are often applied as a chemical solvent for the extraction process of biocatalysts due to the sensitivity of biocatalysts protein structure to pH alterations. Buffers consisting of acetate and citrate at the pH value equal to 4, sodium and potassium phosphate at the pH value equal to 7 and glycine and borax at the pH value equal to 9 are applied as solvent. Additionally, distilled water hexane was selected as non-buffer solvents for biocatalysts extraction. The grinding and mixing were simultaneously performed inside the grinder. Within the extraction process, the size of ground tea leaves was between 0.1 to 1 mm. By passing some certain time, it was filtered and centrifuged at a specific operational condition (11,000 rpm, 4 °C, 30 min). To specify the activity, the filtrate was applied as crude biocatalyst after centrifugation. The desirable operational circumstances for biocatalyst extraction and determination of appropriate solvent are T = 25 °C, buffer pH 4, 7, 9, buffer-to-tea leave ratio = 3, Tea leave and solvent contact time = 2 min, Buffer molarity = 0.05 M, PVP concentration = 0 and Tween 80 concentration = 0^17^.

### Optimization method

RSM procedure (Box-Behnken) is used in this paper to carry out the biocatalysts extraction experiments. The impacts of 7 independent variables consisting of L/S ratio, buffer concentration, addition of surfactant (Tween 80), extraction pH, extraction temperature, addition of PVP, and solid–liquid contact time on the amount of PPO/POD biocatalysts activity are aimed to be investigated. Detailed information of the design is represented in Table 4. All experimental conditions are conducted in triplicate. Design-Expert 11 software is applied to evaluate the statistical analysis.

**Table 4:** Selection of parameters and their levels for RSM (Box-Behnken).

Parameter	Coded Factor and Levels		
	−1	0	1
Temperature, °C	25	50	75
pH	4	7	9
Buffer to biomass ratio	3	5	7
Extraction time, min	2	10	20
Buffer content (M)	0.05	0.1	0.2
PVP content (%)	0	3	6
Tween 80 content (%)	0	5	20

### The measurement of PPO/POD biocatalysts activity

To measure the amount of PPO biocatalyst activity, spectrophotometric method is applied^45^. Catechol solution has been applied as substrate. The sample (cuvette) contains 1.45 ml of 100 mM potassium phosphate buffer with pH of 6.8, 0.5 ml of 100 mM catechol solution, and 0.05 ml of the enzyme solution. The blank sample consists of only 2 ml of substrate solution. The final substrate concentration in the 2 mL assay is 25 mM. The assay mixture is permitted to be incubated for 3 min at room temperature in a disposable polystyrene cuvette^52^. One unit of biocatalyst activity is described as the amount of biocatalyst that results in an alteration in the absorbance of 0.001 per min. PPO activity is assayed in triplicate measurements. Alteration in absorption as a function of time is recorded for 2 min at the ambient temperature. Reaction 3 takes place during assay. As illustrated, the production of this reaction product is *benzoquinone*, which its absorption coefficient is 1010 M^−1^ cm^−1^^45,52^.

POD biocatalyst activity is measured applying *guaiacol* substrate in the presence of hydrogen peroxide at 470 nm. The sample cuvette contains 2.70 mL of 100 mM potassium phosphate buffer with pH value 6.8, 0.15 mL of 4% guaiacol, 0.10 mL of 3% hydrogen peroxide, and 0.05 ml crude biocatalyst^52^. The 4% guaiacol is provided by dispensing 0.4 mL guaiacol in 9.6 mL of deionized water and shaken severely to manufacture an emulsion before application. After incubation, POD activity is monitored at 470 nm for 2 min at room temperature. The reaction product is *biphenoquinone* with the absorption coefficient of 26,600 M^−1^.cm^−1^^45^:

The variation of concentration is able to be predicted on the basis of alteration in the amount of absorbed light. According to Lambert–Beer law, concentration is in direct relationship with absorption value in a wavelength and reverse relationship with thickness of cuvette as the follows^53,54^:

5 $$C=\frac{A}{\varepsilon d}$$

In the abovementioned equation, A, d and ε are respectively interpreted as the amount of absorption, cuvette thickness and molar absorptivity. To evaluate the amounts of PPO/POD activity, the cuvette consisting of biocatalyst was located in the spectrophotometer for 30 s and wavelength alteration with time is recorded. Equation 6 is applied to compute PPO/POD activity^53,54^:

6 $$U=\frac{\Delta C\times V\times {10}^{6}}{\Delta t}=\frac{\Delta A\times V\times {10}^{6}}{\varepsilon \times d\times \Delta t}$$

In this equation, *U*, *V* and *t* are denoted as the biocatalyst activity, the reaction media volume and the reaction time.

### Protein determination

The concentration of protein is determined on the basis of the dye-binding method of Bradford, with bovine serum albumin as standard^55^. The value of obtained absorption is evaluated by graphic interpolation on a calibration curve at 595 nm.

**Figure 5:**
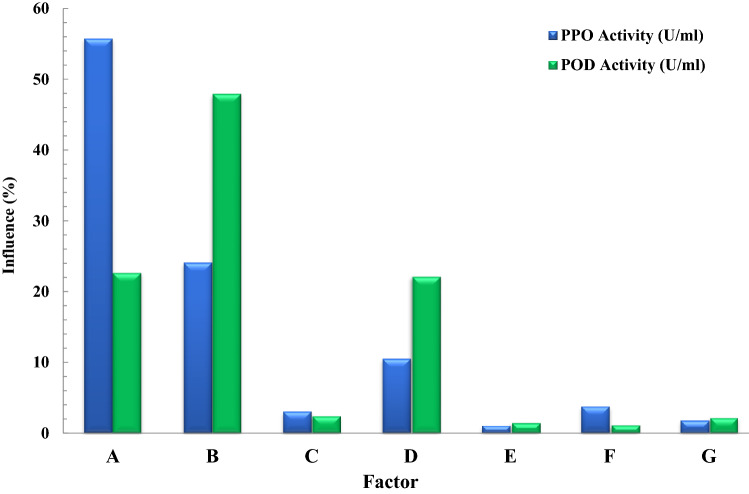
Percentage contribution of each variables on PPO and POD activities.

As shown in Fig. 5, the effect of each of the experimental parameters on the activity of POD and PPO enzymes is determined. The maximum effect on the activity of POD and PPO biocatalysts is the pH and temperature parameters, respectively.

### Evaluation of the heat stability of PPO/POD biocatalysts

Thermal inactivation experiments are carried out in a water bath at constant temperatures of 25, 40, 60 and 80 °C. Samples are exposed to each temperature for 0–60 min and the remaining activity is assayed at 5 min intervals in triplicate. The operational circumstances of PPO/POD biocatalysts extraction applied in thermal inactivation experiment are presented in Table 3.

## Conclusion

Iranian tea leaves are well perceived to possess great potential illustrate PPO and POD activity. RSM (Box-Behnken) method has been employed in this paper to optimize large number of parameters through minimum required tests. By implementing the analysis, final optimum states of extraction factors were obtained. The optimum operational conditions for efficient PPO and POD biocatalysts extraction/activity were; T = 25 °C, pH 7, buffer molarity = 0.1 M, buffer/tea leaves ratio = 5, contact time = 10 min, and presence of 6% PVP and 20% Tween 80 for PPO and T = 50 °C, pH 5, buffer molarity = 0.05 M, buffer/tea leaves ratio = 5, contact time = 10 min, and the existence of 3% PVP and 5% Tween 80 for POD. It was well understood from the research that PPO extracted from tea has the ability of activation in basic media (pH 9). According to the optimization outcomes, the most momentous operational/functional parameters for PPO extraction were temperature (55.67%), pH (24.07%) and addition of Tween 80 (1.79%), while for POD biocatalyst they were pH (47.89%), addition of surfactant (0.01%), and temperature (22.70%). The obtained outcomes from thermal inactivation implied that POD biocatalyst is more resistant compared to PPO. PPO biocatalyst entirely lost its activity after 45 min for all isotherms (other than ambient temperature); while POD maintained its activity even at the temperature of 80 °C (6% at 45 min).

## Supplementary file

Desirability contours of the effect of seven parameters on PPO and POD enzymes activity as well as the calculated activity for PPO and POD enzymes are reported in Supplementary file.

## Supplementary Information

Supplementary Information
