# Supplementary material for: Extraction of ingredients from tea leaves using oxidative enzymatic reaction and optimization of extraction conditions
Source: Sci Rep. 2021 Feb 18;11:4094. doi: 10.1038/s41598-021-83232-x (PMC7892889; doi:10.1038/s41598-021-83232-x)
Supplement: Supplementary file 1 — Supplementary Information [file 41598_2021_83232_MOESM1_ESM.docx]

**(Supporting file)**

**Extraction of Ingredients from Tea Leaves Using Oxidative Enzymatic Reaction and Optimization of Extraction Conditions**

Rasool Pelalak^1,2^, Afrasyab Khan^3^, Masoud Habibi Zare^4^, Mohammad Hasan Sadeghi^4^, Azam Marjani^5,6,*^

^1^ Institute of Research and Development, Duy Tan University, Da Nang 550000, Viet Nam

^2^ Faculty of Environmental and Chemical Engineering, Duy Tan University, Da Nang 550000, Viet Nam

^3^ Institute of Engineering and Technology, Department of Hydraulics and Hydraulic and Pneumatic Systems, South Ural State University (SUSU), Lenin Prospect 76, Chelyabinsk, 454080, Russian Federation

^4^ Isfahan University of Technology, Department of Chemical Engineering, 84156-83111 Isfahan, Iran

^5^ Department for Management of Science and Technology Development, Ton Duc Thang University, Ho Chi Minh City, Viet Nam

^6^ Faculty of Applied Sciences, Ton Duc Thang University, Ho Chi Minh City, Vietnam

^*^Corresponding author; E-mail: [azam.marjani@tdtu.edu.vn](mailto:azam.marjani@tdtu.edu.vn)

| **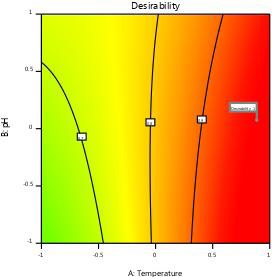** | **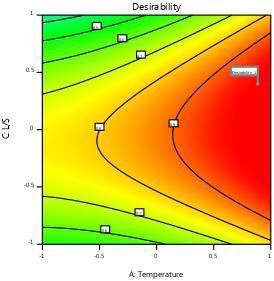** | 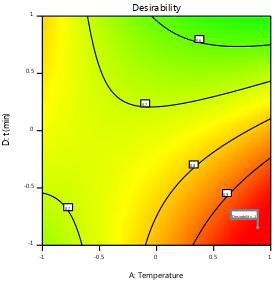 |
| --- | --- | --- |
| **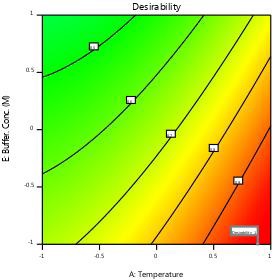** | **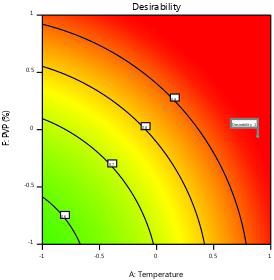** | 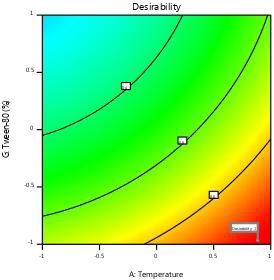 |
| **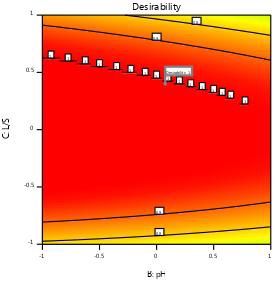** | **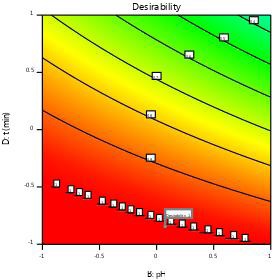** | 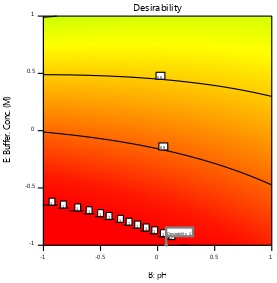 |
| **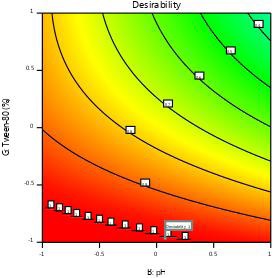** | **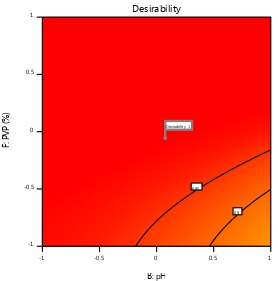** | **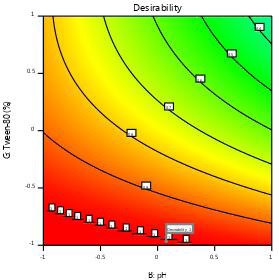** |
| **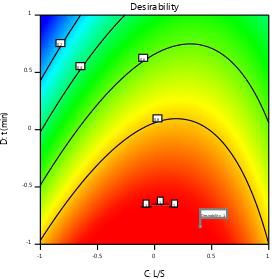** | **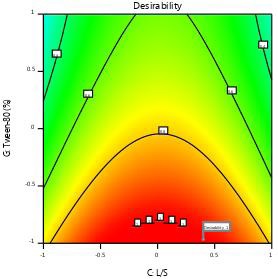** | **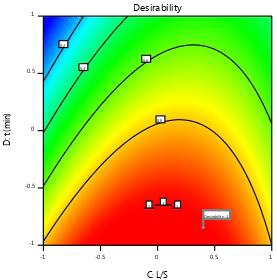** |
| **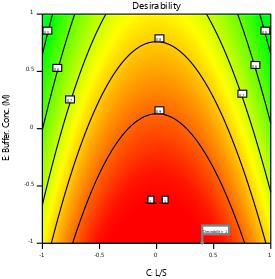** | **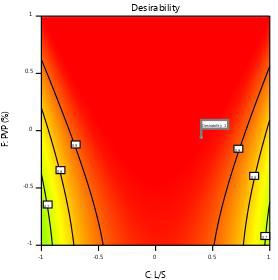** | **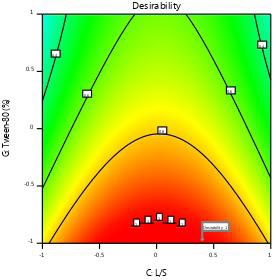** |
| **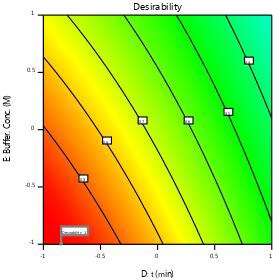** | **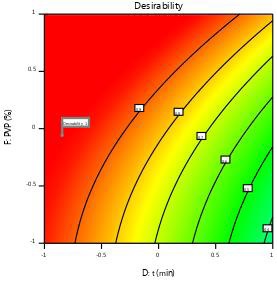** | **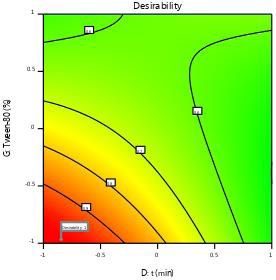** |
| **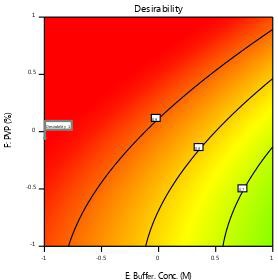** | **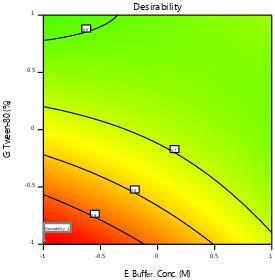** | **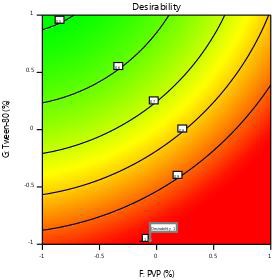** |
| Figure S1: Desirability Contours of the effect of seven parameters on PPO and POD enzymes activity. | | |

Table S1: The calculated activity for PPO enzyme and for POD enzyme.

| Run Order | PPO Activity (U/mL) | | POD Activity (U/mL) | |
| --- | --- | --- | --- | --- |
|  | **Actual Value** | **Predicted Value** | **Actual Value** | **Predicted Value** |
| 1 | 0.2050 | 0.2022 | 1.206E-06 | 1.137E-06 |
| 2 | 0.2961 | 0.3048 | 1.742E-06 | 1.793E-06 |
| 3 | 0.3228 | 0.3352 | 1.899E-06 | 1.930E-06 |
| 4 | 0.3107 | 0.3139 | 1.828E-06 | 1.797E-06 |
| 5 | 0.3101 | 0.3150 | 1.824E-06 | 1.784E-06 |
| 6 | 0.2779 | 0.2801 | 1.634E-06 | 1.718E-06 |
| 7 | 0.1620 | 0.1582 | 9.531E-07 | 1.015E-06 |
| 8 | 0.4197 | 0.4046 | 2.469E-06 | 2.366E-06 |
| 9 | 0.3189 | 0.3048 | 1.876E-06 | 1.793E-06 |
| 10 | 0.2733 | 0.2707 | 1.608E-06 | 1.582E-06 |
| 11 | 0.2170 | 0.2144 | 1.277E-06 | 1.196E-06 |
| 12 | 0.2866 | 0.2815 | 1.686E-06 | 1.759E-06 |
| 13 | 0.2440 | 0.2395 | 1.435E-06 | 1.356E-06 |
| 14 | 0.3921 | 0.3873 | 2.306E-06 | 2.321E-06 |
| 15 | 0.2356 | 0.2407 | 1.386E-06 | 1.420E-06 |
| 16 | 0.2678 | 0.2631 | 1.575E-06 | 1.523E-06 |
| 17 | 0.2779 | 0.2775 | 1.634E-06 | 1.601E-06 |
| 18 | 0.2479 | 0.2425 | 1.458E-06 | 1.425E-06 |
| 19 | 0.2762 | 0.2638 | 1.625E-06 | 1.555E-06 |
| 20 | 0.2860 | 0.2829 | 1.682E-06 | 1.562E-06 |
| 21 | 0.4096 | 0.4047 | 2.410E-06 | 2.386E-06 |
| 22 | 0.2307 | 0.2312 | 1.357E-06 | 1.415E-06 |
| 23 | 0.3257 | 0.3318 | 1.916E-06 | 1.954E-06 |
| 24 | 0.3686 | 0.3690 | 2.168E-06 | 2.248E-06 |
| 25 | 0.2502 | 0.2511 | 1.472E-06 | 1.437E-06 |
| 26 | 0.3338 | 0.3363 | 1.964E-06 | 1.941E-06 |
| 27 | 0.3654 | 0.3659 | 2.549E-06 | 2.249E-06 |
| 28 | 0.2918 | 0.2928 | 1.717E-06 | 1.782E-06 |
| 29 | 0.3761 | 0.3808 | 2.212E-06 | 2.243E-06 |
| 30 | 0.3397 | 0.3406 | 1.998E-06 | 1.901E-06 |
| 31 | 0.1946 | 0.1971 | 1.144E-06 | 1.170E-06 |
| 32 | 0.3602 | 0.3592 | 2.119E-06 | 2.182E-06 |
| 33 | 0.2424 | 0.2452 | 1.426E-06 | 1.576E-06 |
| 34 | 0.3032 | 0.3048 | 1.784E-06 | 1.793E-06 |
| 35 | 0.2281 | 0.2369 | 1.342E-06 | 1.340E-06 |
| 36 | 0.2382 | 0.2414 | 1.401E-06 | 1.445E-06 |
| 37 | 0.2700 | 0.2676 | 1.589E-06 | 1.611E-06 |
| 38 | 0.3058 | 0.3050 | 1.799E-06 | 1.824E-06 |
| 39 | 0.2453 | 0.2508 | 1.443E-06 | 1.477E-06 |
| 40 | 0.2564 | 0.2559 | 1.508E-06 | 1.469E-06 |
| 41 | 0.2915 | 0.2897 | 1.715E-06 | 1.718E-06 |
| 42 | 0.2144 | 0.2189 | 1.261E-06 | 1.353E-06 |
| 43 | 0.2993 | 0.3048 | 1.761E-06 | 1.793E-06 |
| 44 | 0.3937 | 0.3984 | 2.316E-06 | 2.328E-06 |
| 45 | 0.3384 | 0.3337 | 1.990E-06 | 1.966E-06 |
| 46 | 0.1767 | 0.1805 | 1.039E-06 | 1.014E-06 |
| 47 | 0.2941 | 0.3093 | 1.730E-06 | 1.897E-06 |
| 48 | 0.2502 | 0.2528 | 1.472E-06 | 1.564E-06 |
| 49 | 0.3039 | 0.3048 | 1.788E-06 | 1.793E-06 |
| 50 | 0.1724 | 0.1742 | 1.014E-06 | 1.011E-06 |
| 51 | 0.2352 | 0.2343 | 1.384E-06 | 1.395E-06 |
| 52 | 0.3075 | 0.3048 | 1.809E-06 | 1.793E-06 |
| 53 | 0.3055 | 0.3050 | 1.797E-06 | 1.925E-06 |
| 54 | 0.2905 | 0.2817 | 1.709E-06 | 1.692E-06 |
| 55 | 0.4109 | 0.4077 | 2.417E-06 | 2.451E-06 |
| 56 | 0.3800 | 0.3777 | 2.235E-06 | 2.167E-06 |
| 57 | 0.1571 | 0.1510 | 9.244E-07 | 8.865E-07 |
| 58 | 0.4034 | 0.4043 | 2.373E-06 | 2.345E-06 |
| 59 | 0.2040 | 0.2007 | 1.200E-06 | 1.144E-06 |
| 60 | 0.3283 | 0.3314 | 1.931E-06 | 1.957E-06 |
| 61 | 0.3878 | 0.3869 | 2.281E-06 | 2.315E-06 |
| 62 | 0.3400 | 0.3448 | 2.000E-06 | 1.984E-06 |
